# Opinion of Polish doctors on the use of futile therapy

**DOI:** 10.1093/eurpub/ckae202

**Published:** 2024-12-20

**Authors:** Maria Damps, Maksymilian Gajda, Łukasz Wiktor, Elżbieta Byrska-Maciejasz, Beata Rybojad, Małgorzata Kowalska, Alicja Bartkowska-Śniatkowska, Anna Paprocka-Lipińska, Ewa Kucewicz-Czech

**Affiliations:** Department of Anesthesiology and Intensive Care, Upper Silesian Child Health Centre, Faculty of Medical Sciences in Katowice, Medical University of Silesia, Katowice, Poland; Department of Epidemiology, School of Medicine in Katowice, Medical University of Silesia, Katowice, Poland; Department of Trauma and Orthopedic Surgery, Upper Silesian Children’s Health Centre, Medical University of Silesia, Katowice, Poland; Department of Anesthesiology and Intensive Therapy, University Children’s Hospital of Krakow, Krakow, Poland; Department of Anesthesiology and Pediatric Intensive Care, Faculty of Medicine, Medical University of Lublin, Lublin, Poland; Department of Epidemiology, School of Medicine in Katowice, Medical University of Silesia, Katowice, Poland; Department of Pediatric Anesthesiology and Intensive Care, Poznań University of Medical Sciences, Poznań, Poland; Department of Medical Ethics, Medical University of Gdansk, Gdansk, Poland; Department of Anesthesiology and Intensive Therapy, Silesian Centre for Heart Diseases in Zabrze, Medical University of Silesia, Zabrze, Poland

## Abstract

The discontinuation of futile therapy is increasingly discussed in Polish clinical practice. Given the need to ensure patient well-being, it is essential to consider whether all clinical options resulting from medical progress should be used for every patient and on what grounds decisions to limit therapy should be based. The aim of our study was to determine the opinions of Polish medical doctors on this topic. We anonymously surveyed physicians across various specialties. An analysis of the collected data was carried out using descriptive and analytical methods. A total of 323 physicians participated in the study; 93% of them were aware of the problem of futile therapy in adults, with intensivists being significantly more aware (*P* = 0.002). Additionally, 95% of respondents supported the idea of discontinuing futile therapy, and over 68% used the therapy discontinuation protocol. Among the most common reasons for undertaking futile therapy, respondents cited fear of legal liability (93.5%), as well as fear of being accused of unethical behavior (62.2%) and fear before talking to the patient/patient’s family and their reactions (57.9%). Respondents also identified factors that would facilitate making decisions about limiting futile therapy, including precise qualification criteria for limiting therapy and education in this area (95.3%), the patient’s declaration of will (87.5%), and a clear legal act (81.3%). The majority of study participants supported the idea of limiting futile therapy, and this issue is well known among Polish physicians.

## Introduction

Polish research on therapeutic boundaries was initiated by experts in the fields of ethics, medicine, and law at a conference entitled “The limits of medical therapies” in 2008. The tangible result of this scientific meeting was the establishment of the Polish Working Group on End-of-Life Ethical Problems and the development of a definition of futile medical care [1]. Maintaining the functions of organs that do not bring any benefits to the patient and do not make it possible to achieve the assumed therapeutic goals is called futile therapy (FT). FT prolongs the dying process and is related to the suffering of patients and their families, and the violation of human dignity. This definition is similar to the term “low-value care”—unlikely to benefit patients and can even unintentionally harm them [2]. However, FT is a broader medical-ethical-legal concept. Limiting FT requires palliative treatment, ensuring the patient’s comfort and providing care, nutrition, and hydration. Based on this definition, recommendations and guidelines for making medical decisions regarding medically futile actions were developed. The first recommendations for discontinuing persistent life-sustaining treatment were published for pediatricians (2011) [3] followed by specialists in anesthesiology and intensive care of adults (2014) [4], neonatologists (2019) [5], and pediatric intensive care unit (PICU) specialists (2022) [6]. Based on the position of the appointed team of experts, the Patient Ombudsman issued a publication on the implementation of the patient’s right to a dignified death, containing standards for end-of-life medical therapies (2021) [7]. The latest Polish document extensively discussing end-of-life medical care is the Position of the Polish Society of Internal Medicine Working Group on Medical Futility at Internal Medicine Units [8].

Physicians providing care to critically and terminally ill patients have received useful tools to assist in making ethically significant decisions.

Considering the broadly understood well-being of patients, it is necessary to evaluate whether all clinical options resulting from medical progress should be applied to every patient and to establish criteria for deciding when to limit therapy. The SarsCoV-2 pandemic necessitated reconsideration of triage protocols and the distribution of resources, particularly funding [9].

The debate on the use/limitation of FT in our country is taking place behind the scenes. Apart from the above-mentioned guidelines and recommendations of scientific societies, we still do not know the real independent opinions of medical doctors, nurses, and society on this subject. Therefore, we decided to conduct a questionnaire survey among medical doctors, nurses, and students (as representatives of the society) to learn about the clinical practices and personal opinions of the respondents on the discussed topic. We believe, that the obtained results of the study help explain the perception of the mentioned problem, recognize its determinants as well as indicate possible solutions, and determine further procedures to improve the clinical and social situation. Therefore, a survey was designed to determine the opinions of physicians, nurses, and students. The results regarding the opinions of Polish nurses have already been analyzed and published [10]. In this study, we present the results of the survey conducted among physicians. Further research assessing public opinions on this topic is in progress. The purpose of our work was to understand the clinical practice and opinions of Polish medical doctors regarding the use of FT. We aimed to answer the significant question: How often is FT used, why is it used, and what can be done to change this practice?

## Methods

In 2022, a cross-sectional study was conducted using a proprietary questionnaire (Supplementary Material). The Polish-language questionnaire consisted of two parts with a total of 26 questions. The first part consisted of eight questions focused on sociodemographic characteristics, such as sex, age, and religion, as well as professional information, including practice, workplace, and medical specialties. In Poland, anesthesiology and intensive care remain joint specialties—physicians in this field both administer anesthesia in operating rooms and manage intensive care units (ICUs), with no official separation between pediatric and adult specialists. The second part of the survey consisted of 18 questions focused on medical workers’ opinions on various aspects of FT, including the reasons for its implementation and the legitimacy of its limitations. The questionnaire was available in two forms, traditional paper and electronic, and contained both open-ended and closed questions (single- and multiple-choice). All respondents were asked to answer the questions on their own. Electronic access was secured by a professional license number and e-mail verification. The questions were prepared by the authors of the study after consultation with Polish experts in this field such as medical doctors with extensive experience in ICUs, specialists in palliative medicine, or representatives of the Polish Palliative Scientific Society. The questionnaire was validated in a group of 20 randomly selected medical workers, who completed the survey twice, 1 week apart. The percentage agreement of the answers between the test and retest was assessed, and Cohen’s kappa was calculated for the key questions. The obtained results indicated good or very good repeatability of the questions. Physicians of all specialties employed in hospitals located in the Silesian, Lesser Poland, Subcarpathian, and Greater Poland voivodeships were invited to participate in the study (500 subjects, representing approximately 4% of professionally active physicians in these regions). Participation was voluntary and anonymous; study participants did not incur additional costs or receive any compensation. The study design was approved by the Ethics Committee of the Medical University of Silesia (KNW/0022/KB/284/18). We confirm that all methods were performed in accordance with the relevant guidelines and regulations.

The received paper surveys were checked for completeness. The study included questionnaires with a full answer of eight questions focused on sociodemographic characteristics and at least 16 of the 18 questions of the second part. Eleven surveys out of 142 paper versions were rejected because of the lack of completed data. In the electronic version, the system required answers to all questions. Depending on the type of question, the answers to open-ended questions were grouped by the principal investigator and the author of the survey and matched to the simplified categories. We differentiated the answers between intensivists and non-intensivists due to the significant difference in practical experience regarding the discussed topic. We were curious whether the opinion of medical doctors not working in ICUs differed significantly from that of intensivists. Since the limitation of FT in children is very controversial, we wanted to check whether the subjects’ declaration varies on the type of patients, adults or children. Qualitative variables were characterized as numbers and percentages. The χ^2^ test of independence was used to determine whether there were significant relationships between pairs of nominal variables. As quantitative variables deviated from the normal distribution, the Shapiro–Wilk test was used and reported as the median with the interquartile range (IQR). For nonparametric tests, the Mann–Whitney *U* test was used to analyze differences between groups. In all analyses, the criterion of *P* < 0.05 was used to indicate statistical significance. All statistical analyses were performed using R software (version 4.1.0, R Foundation for Statistical Computing, Vienna, Austria).

## Results

### Characteristics of the study population

Among the 500 physicians who were invited to participate, 323 joined the study (64.6% participation rate). The paper version was completed by 131 respondents (40.6%) and the online version was completed by 192 participants (59.4%). The median age of the participants was 40 years (IQR 33–51), and intensivists were significantly older than other specialists (42 vs. 37 years, *P* = 0.017). The majority of the study participants were women (*N* = 211, 65.3%) and Catholics (70.2%); however, 57% of the respondents reported weekly participation in church services (see Table 1).

**Table 1.** General characteristics of the studied population

		Overall *n* (%)	Intensivists *n* (%)		
		*n* = 323	No 140 (43.34%)	Yes 183 (56.66%)	*P*
Mode of interview	Offline	131 (40.6%)	53 (37.9%)	78 (42.6%)	0.688
	Online	192 (59.4%)	87 (62.1%)	105 (57.4%)	
Age (years)	Median [q25–q75]	40.0 [33.0–51.0]	37.0 [31.0–48.0]	42.0 [35.0–53.0]	**0.017**
	Missing	2 (0.6%)	1 (0.7%)	1 (0.5%)	
Sex	Female	211 (65.3%)	101 (72.1%)	110 (60.1%)	0.079
	Male	112 (34.7%)	39 (27.9%)	73 (39.9%)	
Length of employment (years)	Median [q25–q75]	14.0 [7.00–25.0]	12.0 [5.00–23.0]	15.0 [8.50–26.0]	0.052
Work in the intensive care unit	Yes	184 (58.4%)	27 (19.9%)	157 (87.7%)	**<0.001**
	No	131 (41.6%)	109 (80.1%)	22 (12.3%)	
	Missing	8 (2.5%)	4 (2.9%)	4 (2.2%)	
Place of work—teaching hospital with ICU	Yes	184 (57.0%)	88 (62.9%)	96 (52.5%)	0.174
	No	139 (43.0%)	52 (37.1%)	87 (47.5%)	
Place of work—nonclinical ICU hospital	Yes	184 (57.0%)	88 (62.9%)	96 (52.5%)	0.174
	No	139 (43.0%)	52 (37.1%)	87 (47.5%)	
Place of work—hospital without ICU	Yes	184 (57.0%)	88 (62.9%)	96 (52.5%)	0.174
	No	139 (43.0%)	52 (37.1%)	87 (47.5%)	
Confession	Catholic who attends church every week	129 (40.1%)	65 (46.4%)	64 (35.2%)	0.173
	Catholic	97 (30.1%)	31 (22.1%)	66 (36.3%)	
	Atheist	67 (20.8%)	33 (23.6%)	34 (18.7%)	
	Protestant, Baptist	29 (9.0%)	11 (7.9%)	18 (9.9%)	
	Missing	1 (0.3%)	0 (0%)	1 (0.5%)	

Bold values represent statistically significant.

### Work experience and workplace

The median work experience was 14 years (IQR 7–25 years), which did not differ between intensivists and other specialists. Among non-intensivists, 19.9% of participants had experience working in ICUs. Notably, most of the respondents were employed in university hospitals with ICUs (57%; see Table 1).

### Experience and opinions related to futile therapy

Less than half (40%) of the respondents talked to patients’ families about the discontinuation of therapy, with no differences found between specialties. Additionally, 41.5% of respondents (*N* = 117) reported feeling prepared to have these conversations with their patients with intensivists feeling significantly more prepared than other specialties (82% vs. 35%, *P* = 0.01). However, 30% of participants did not consider themselves prepared for such a conversation, regardless of their specialty, and 6% of physicians avoided talking to patients’ families despite the need to do so. The majority of the respondents who reported having no experience talking to patients’ families about this topic did not work in a place where such a need existed. Additionally, one question on the survey addressed the frequency of possible contact with a dying patient. More than half of the respondents (60.1%) declared that such an opportunity occurred several times a month, with intensivists having the greatest opportunity for contact. Most of the respondents (93%) were familiar with the problem of FT in adults, with intensivists being significantly more aware of it (*P* = 0.002). Additionally, more than 73% of respondents had experience with FT in pediatric practice, with no significant differences between specialties. Over 87% of the surveyed intensivists knew the guidelines for discontinuing FT in adults, whereas only about half were aware of similar documentation for children. A total of 95% of respondents supported the idea of discontinuing FT, and over 68% of respondents had used the therapy discontinuation protocol (see Table 2).

**Table 2.** Work experience and physicians’ views on futile therapy (numbers and percentages in brackets)

	Overall *n* (%) 323	Intensivists *n* (%)		*P*
		No 140 (43.34%)	Yes 183 (56.66%)	
Contact with a dying patient				
Few cases in recent years	42 (13.0%)	37 (26.4%)	5 (2.7%)	**<0.001**
Several cases a year	87 (26.9%)	56 (40.0%)	31 (16.9%)	
Several cases a month	194 (60.1%)	47 (33.6%)	147 (80.3%)	
Witness to illness and death				
Yes	238 (73.9%)	90 (64.7%)	148 (80.9%)	**0.005**
No	84 (26.1%)	49 (35.3%)	35 (19.1%)	
Missing	1 (0.3%)	1 (0.7%)	0 (0%)	
Knowledge of futile therapy in adults				
Yes	297 (93.7%)	121 (88.3%)	176 (97.8%)	**0.003**
No	20 (6.3%)	16 (11.7%)	4 (2.2%)	
Missing	6 (1.9%)	3 (2.1%)	3 (1.6%)	
Knowledge of futile therapy in children				
Yes	228 (71.3%)	100 (71.9%)	128 (70.7%)	0.972
No	92 (28.8%)	39 (28.1%)	53 (29.3%)	
Missing	3 (0.9%)	1 (0.7%)	2 (1.1%)	
Knowledge of guidelines for futile therapy				
Yes	206 (64.4%)	47 (33.8%)	159 (87.8%)	**<0.001**
No	114 (35.6%)	92 (66.2%)	22 (12.2%)	
Missing	3 (0.9%)	1 (0.7%)	2 (1.1%)	
Talk to patients and/or their family about discontinuing futile therapy				
Yes	134 (41.5%)	61 (43.6%)	73 (39.9%)	0.966
No, even though it was necessary	21 (6.5%)	8 (5.7%)	13 (7.1%)	
No, due to the type of specialization	168 (52.0%)	71 (50.7%)	97 (53%)	
Prepared for discussing futile therapy discontinuation with the patient and/or the patient’s family				
Yes	117 (41.5%)	35 (30.7%)	82 (48.8%)	**0.014**
No opinion	80 (28.4%)	44 (38.6%)	36 (21.4%)	
No	85 (30.1%)	35 (30.7%)	50 (29.8%)	
Missing	41 (12.7%)	26 (18.6%)	15 (8.2%)	
Use of futile therapy is a mistake				
Yes	187 (57.9%)	76 (54.3%)	111 (60.7%)	0.103
No opinion	59 (18.3%)	35 (25.0%)	24 (13.1%)	
No	77 (23.8%)	29 (20.7%)	48 (26.2%)	
Belief that discontinuing futile therapy is right				
Yes	306 (94.7%)	132 (94.3%)	174 (95.1%)	0.257
No opinion	10 (3.1%)	7 (5.0%)	3 (1.6%)	
No	7 (2.2%)	1 (0.7%)	6 (3.3%)	
Belief that economic aspects should be decisive in discontinuation of futile therapy				
Yes	130 (40.2%)	55 (39.3%)	75 (41.0%)	0.957
No opinion	56 (17.3%)	27 (19.3%)	29 (15.8%)	
No	137 (42.4%)	58 (41.4%)	79 (43.2%)	
What would make the decision to limit futile therapy easier?				
Unambiguous legal act				
Yes	260 (81.3%)	105 (75.0%)	155 (86.1%)	0.138
Don’t know	37 (11.6%)	23 (16.4%)	14 (7.8%)	
No	23 (7.2%)	12 (8.6%)	11 (6.1%)	
Missing	3 (0.9%)	0 (0%)	3 (1.6%)	
Precise eligibility criteria for limiting therapy				
Yes	305 (95.3%)	133 (96.4%)	172 (94.5%)	0.947
Don’t know	8 (2.5%)	3 (2.2%)	5 (2.7%)	
No	7 (2.2%)	2 (1.4%)	5 (2.7%)	
Missing	3 (0.9%)	2 (1.4%)	1 (0.5%)	
Education in this area (practical form of education, e.g. workshops)				
Yes	307 (95.3%)	130 (92.9%)	177 (97.3%)	0.25
Don’t know	8 (2.5%)	4 (2.9%)	4 (2.2%)	
No	7 (2.2%)	6 (4.3%)	1 (0.5%)	
Missing	1 (0.3%)	0 (0%)	1 (0.5%)	
Patient’s declaration of will/living will				
Yes	279 (87.5%)	119 (86.2%)	160 (88.4%)	0.333
Don’t know	25 (7.8%)	15 (10.9%)	10 (5.5%)	
No	15 (4.7%)	4 (2.9%)	11 (6.1%)	
Missing	4 (1.2%)	2 (1.4%)	2 (1.1%)	

Bold values represent statistically significant.

### Reasons for futile therapy

The main reason for the family’s refusal to limit FT was not accepting the inevitability of death (Table 3). Among the most common reasons for continuing FT in adult patients, respondents cited fears of legal liability for not taking such actions (93.5%), concerns about being accused of unethical behavior (62.2%), and fears of discussing the situation with the patient or their family and their reactions (57.9%). While non-intensivists were more likely to be afraid of the opinions of their colleagues, they also believed that one must fight heroically for life until the very end. The opinions of the respondents regarding working with pediatric patients were similar, but the fear of talking to parents and being accused of unethical behavior predominated. Nearly half of the respondents noted that the reason for using FT was the recommendation of the head of the department (see Table 3).

**Table 3.** Reasons for starting futile therapy in adult and pediatric patients

	Overall *n* (%)	Intensivists *n* (%)		*P*
		No 140 (43.34%)	Yes 183 (56.66%)	
Main reasons for undertaking/continuing futile therapy in adult patients:				
Fear of talking to the patient/patient’s family and their reaction				
Yes	187 (57.9%)	86 (61.4%)	101 (55.2%)	0.531
No	136 (42.1%)	54 (38.6%)	82 (44.8%)	
Fear of legal liability for withdrawing or withholding treatment				
Yes	302 (93.5%)	129 (92.1%)	173 (94.5%)	0.688
No	21 (6.5%)	11 (7.9%)	10 (5.5%)	
Heroic fight for life to the end, because this is the ethical duty of the doctor				
Yes	82 (25.4%)	50 (35.7%)	32 (17.5%)	**<0.001**
No	241 (74.6%)	90 (64.3%)	151 (82.5%)	
Fear of being accused by the patient’s family of a lack of professional ethics				
Yes	65 (20.1%)	39 (27.9%)	26 (14.2%)	**0.010**
No	258 (79.9%)	101 (72.1%)	157 (85.8%)	
Fear of being accused by the patient’s family of the lack of professional ethics				
Yes	201 (62.2%)	97 (69.3%)	104 (56.8%)	0.073
No	122 (37.8%)	43 (30.7%)	79 (43.2%)	
Order/recommendation from the supervisor				
Yes	126 (39.0%)	49 (35.0%)	77 (42.1%)	0.434
No	197 (61.0%)	91 (65.0%)	106 (57.9%)	
Passivity in action (colloquial term—laziness)				
Yes	81 (25.1%)	22 (15.7%)	59 (32.2%)	**0.003**
No	242 (74.9%)	118 (84.3%)	124 (67.8%)	
Main reasons for undertaking/continuing futile therapy in newborns/children				
Fear of talking to the patient/patient’s parents (family) and their reaction				
Yes	243 (75.2%)	105 (75.0%)	138 (75.4%)	0.996
No	80 (24.8%)	35 (25.0%)	45 (24.6%)	
Fear of legal liability for withdrawing or withholding treatment				
Yes	300 (92.9%)	127 (90.7%)	173 (94.5%)	0.417
No	23 (7.1%)	13 (9.3%)	10 (5.5%)	
Heroic fight for life to the end, because this is the ethical duty of the doctor				
Yes	171 (52.9%)	88 (62.9%)	83 (45.4%)	**0.007**
No	152 (47.1%)	52 (37.1%)	100 (54.6%)	
Fear of being accused by colleagues of a lack of professional ethics				
Yes	95 (29.4%)	49 (35.0%)	46 (25.1%)	0.156
No	228 (70.6%)	91 (65.0%)	137 (74.9%)	
Fear of being accused by the patient’s family of a lack of professional ethics				
Yes	218 (67.5%)	108 (77.1%)	110 (60.1%)	**0.005**
No	105 (32.5%)	32 (22.9%)	73 (39.9%)	
Order/recommendation from the supervisor				
Yes	138 (42.7%)	55 (39.3%)	83 (45.4%)	0.55
No	185 (57.3%)	85 (60.7%)	100 (54.6%)	
Passivity in action (colloquial term—laziness)				
Yes	65 (20.1%)	22 (15.7%)	43 (23.5%)	0.224
No	258 (79.9%)	118 (84.3%)	140 (76.5%)	

Bold values represent statistically significant.

### Limitations of futile therapy and related decisions

The majority of respondents (94.7%) believed that limiting FT was appropriate, but more than half (57.9%) considered the use of FT as a medical error.

One-fifth of the respondents had no opinion on this topic. Opinions on the impact of economic considerations on the use or discontinuation of FT were equally divided. Among the reasons for the lack of consent of the patient’s family to limit FT, the most common was a lack of acceptance of the inevitability of death (*n* = 255, 78.9%). Almost half of respondents (157, 48.6%) indicated this was related to patient’s family members’ belief in the supernatural possibilities of medicine. Almost 60% of the surveyed physicians believed that the family did not trust them enough, which was why they did not agree to discontinue therapy.

The respondents also reported on factors regarding making decisions about limiting FT. These included precise qualification criteria for limiting therapy and education in this area (95.3%), the patient’s declaration of will (87.5%), and a clear legal framework (81.3%; see Table 2).

A crucial goal was to identify the entity responsible for the discontinuation of FT. In the case of adult patients, respondents believed that the cessation of FT should be decided by the patient in a declaration of will (94.1%), followed by the physician (97.2%). Approximately one-third of respondents mentioned the patient’s family, except for intensivists, who reported the patient’s significantly less frequently than other specialties. In controversial cases, almost 40% of respondents indicated the court to be the entity responsible for making the decision to discontinue FT. In the case of FT among pediatric patients, 95.7% of the physicians indicated medical rationale as the reason for discontinuation. Decisions made by parents were indicated by 65.2% of the respondents as the reason for discontinuation, and approximately half of the respondents indicated the court. Notably, 92.9% of the physicians agreed to withdraw futile treatment if the patient suffered from an incurable disease. Most participants (93.1%) stated that the decision to discontinue FT should be made by the patient in a living will or a declaration of will. Over 90% of the respondents indicated the decision should be made by the physician and approximately 40% reported it should be determined by the family, with approximately a quarter of respondents indicating the decision should be made by the court (see Table 4).

**Table 4.** Physician’s opinions on who should decide to discontinue futile therapy in adult and pediatric patients

	Overall *n* (%) 323	Intensivists		*P*
		No 140 (43.34%)	Yes 183 (56.66%)	
Who should decide to discontinue futile therapy in adult patients?				
Patient in a declaration of will/living will				
Yes	301 (94.1%)	131 (94.9%)	170 (93.4%)	0.948
Don’t know	9 (2.8%)	4 (2.9%)	5 (2.7%)	
No	10 (3.1%)	3 (2.2%)	7 (3.8%)	
Missing	3 (0.9%)	2 (1.4%)	1 (0.5%)	
Doctors				
Yes	314 (97.2%)	133 (95.0%)	181 (98.9%)	0.343
Don’t know	4 (1.2%)	3 (2.1%)	1 (0.5%)	
No	5 (1.5%)	4 (2.9%)	1 (0.5%)	
Nurses				
Yes	43 (14.4%)	14 (10.8%)	29 (17.3%)	0.239
Don’t know	68 (22.8%)	37 (28.5%)	31 (18.5%)	
No	187 (62.8%)	79 (60.8%)	108 (64.3%)	
Missing	25 (7.7%)	10 (7.1%)	15 (8.2%)	
Patient’s family				
Yes	112 (36.6%)	62 (45.9%)	50 (29.2%)	**0.004**
Don’t know	54 (17.6%)	28 (20.7%)	26 (15.2%)	
No	140 (45.8%)	45 (33.3%)	95 (55.6%)	
Missing	17 (5.3%)	5 (3.6%)	12 (6.6%)	
Court				
Yes	118 (38.4%)	60 (44.4%)	58 (33.7%)	0.389
Don’t know	70 (22.8%)	30 (22.2%)	40 (23.3%)	
No	119 (38.8%)	45 (33.3%)	74 (43.0%)	
Missing	16 (5.0%)	5 (3.6%)	11 (6.0%)	
Who should decide to discontinue futile therapy in pediatric patients?				
Doctors				
Yes	309 (95.7%)	132 (94.3%)	177 (96.7%)	0.469
Don’t know	6 (1.9%)	2 (1.4%)	4 (2.2%)	
No	8 (2.5%)	6 (4.3%)	2 (1.1%)	
Head physician				
Yes	200 (64.7%)	79 (59.0%)	121 (69.1%)	0.474
Don’t know	41 (13.3%)	20 (14.9%)	21 (12.0%)	
No	68 (22.0%)	35 (26.1%)	33 (18.9%)	
Missing	14 (4.3%)	6 (4.3%)	8 (4.4%)	
Nurses				
Yes	41 (13.8%)	13 (10.0%)	28 (16.8%)	0.573
Don’t know	68 (22.9%)	30 (23.1%)	38 (22.8%)	
No	188 (63.3%)	87 (66.9%)	101 (60.5%)	
Missing	26 (8.0%)	10 (7.1%)	16 (8.7%)	
Child’s parents				
Yes	204 (65.2%)	95 (69.3%)	109 (61.9%)	0.253
Don’t know	35 (11.2%)	18 (13.1%)	17 (9.7%)	
No	74 (23.6%)	24 (17.5%)	50 (28.4%)	
Missing	10 (3.1%)	3 (2.1%)	7 (3.8%)	
Guardianship/court				
Yes	146 (47.9%)	72 (53.3%)	74 (43.5%)	0.503
Don’t know	63 (20.7%)	27 (20.0%)	36 (21.2%)	
No	96 (31.5%)	36 (26.7%)	60 (35.3%)	
Missing	18 (5.6%)	5 (3.6%)	13 (7.1%)	
Missing	3 (0.9%)	2 (1.4%)	1 (0.5%)	
Who would decide to discontinue futile therapy in relation to the surveyed				
He or she				
Yes	295 (93.1%)	131 (94.9%)	164 (91.6%)	0.816
Don’t know	14 (4.4%)	5 (3.6%)	9 (5.0%)	
No	8 (2.5%)	2 (1.4%)	6 (3.4%)	
Missing	6 (1.9%)	2 (1.4%)	4 (2.2%)	
Doctors				
Yes	294 (92.7%)	126 (90.6%)	168 (94.4%)	0.64
Don’t know	7 (2.2%)	5 (3.6%)	2 (1.1%)	
No	16 (5.0%)	8 (5.8%)	8 (4.5%)	
Missing	6 (1.9%)	1 (0.7%)	5 (2.7%)	
Head physician				
Yes	154 (50.7%)	63 (47.4%)	91 (53.2%)	0.761
Don’t know	44 (14.5%)	18 (13.5%)	26 (15.2%)	
No	106 (34.9%)	52 (39.1%)	54 (31.6%)	
Missing	19 (5.9%)	7 (5.0%)	12 (6.6%)	
Nurses				
Yes	35 (11.8%)	11 (8.4%)	24 (14.5%)	0.565
Don’t know	41 (13.8%)	17 (13.0%)	24 (14.5%)	
No	221 (74.4%)	103 (78.6%)	118 (71.1%)	
Missing	26 (8.0%)	9 (6.4%)	17 (9.3%)	
Family				
Yes	121 (39.7%)	62 (45.9%)	59 (34.7%)	0.288
Don’t know	36 (11.8%)	17 (12.6%)	19 (11.2%)	
No	148 (48.5%)	56 (41.5%)	92 (54.1%)	
Missing	18 (5.6%)	5 (3.6%)	13 (7.1%)	
Court				
Yes	69 (23.0%)	36 (27.1%)	33 (19.8%)	0.689
Don’t know	56 (18.7%)	23 (17.3%)	33 (19.8%)	
No	175 (58.3%)	74 (55.6%)	101 (60.5%)	
Missing	23 (7.1%)	7 (5.0%)	16 (8.7%)	

Bold value represent statistically significant.

### Differences in the opinions of intensivists

The intensivists who participated in the study had greater knowledge of the discontinuation of FT, relevant recommendations, and guidelines as well as more practical experience in caring for dying patients. In addition, intensivists were much less likely to allow the patient’s family to make the decision to discontinue the patient’s therapy (*P* = 0.004). Intensivists were also less concerned about the opinions of other doctors and the patient’s family regarding the ethics of their behavior (*P* < 0.001 and *P* = 0.005, respectively). They did not feel a heroic obligation to provide futile treatment by fighting to the end (*P* = 0.007) and more frequently admitted to passivity in action, such as avoiding making decisions (*P* = 0.003). Additionally, intensivists considered the use of renal replacement therapy as a component of FT (*P* < 0.001), similar to pharmacological and mechanical support of the circulatory system (*P* = 0.03).

## Discussion

This research shows that the issue of FT is widely known in the Polish medical community, and the majority of respondents consider its use to be medical malpractice. Similar results were obtained in a study conducted in Poland in 2011 by Kübler *et al.* [11]. Among physicians they assessed working in ICUs, 93% reported not undertaking certain therapeutic activities, and 75% refrained from other activities due to their futility. Although Polish law allows the discontinuation of therapy, the matter is still not regulated. For most of the surveyed doctors, a clear legal act, if introduced, would facilitate decision-making in coordination with the already published Guidelines and the Position of the Patient Ombudsman. In a study conducted in 2016 among Australian physicians, one-third of the respondents indicated fear of legal consequences as the reason for conducting FT [12]. The Worldwide End-of-Life Practice for Patients in Intensive Care Units (WELPICUS) [13], collected opinions from 178 hospitals in 31 countries and found that the individual factors that had the greatest impact on decision-making regarding end-of-life care were doctors’ beliefs, hospital policies, and regional conditions. However, in this study, approximately 80% of the physicians supported the idea of withholding and withdrawing therapeutic procedures due to their futility, regardless of an agreement with the patient's family.

On the other hand, Japanese ethicists leave the decision to the family and note the need for honest information from medical staff about the futility of therapy [14]. The difference in opinions highlights not only the role of the regional culture but also the fact that the discussion conducted in the humanist community may differ significantly from that in the medical community. Nevertheless, proper communication with the patient's family plays an important role in the process of avoiding FT [15–17]. Our respondents indicated that the primary reasons for continuing FT included fear of talking to a family that does not accept the inevitability of the patient’s death, lack of trust in the doctor, and concern about potential legal claims from the family. In our previous study regarding nurses' opinions on the discussed topic [10], 6% of participants believed that they were sufficiently prepared for such a conversation. This issue requires comprehensive education, workshops, and the development of soft skills, as indicated by over 90% of our survey participants. Simply believing in the validity of discontinuing therapy is not sufficient to improve the quality of end-of-life care.

A declaration of will, i.e. the willingness to decide for oneself, is the most popular proposed solution to controversial situations [18–21]. Respecting the patient’s will and self-determination regarding their treatment would, in the authors’ opinion, be an expression of respect for the patient’s right to make decisions. However, patients often lack influence over their end-of-life care. The concept of a living will also poses many practical problems. It is impossible to predict future and uncertain events. Therefore, in such cases, information about the nature, purpose, and effects of medical interventions may not be complete. Opponents of living wills also argue that perceptions of quality of life and the will to live can differ significantly between younger and older individuals. Moreover, a living will needs to be constantly updated. Another possible solution is to introduce a medical proxy [22, 23]. However, we did not ask about the institution of a medical proxy, which is a limitation of our study. To the best of our knowledge, there is poor knowledge on this subject in the Polish medical community, but we see a need to deepen and investigate this issue in future research.

Many of our respondents highlighted the particular difficulty of talking to the parents of pediatric patients. They also expressed feelings of ethical responsibility to ensure that medical decisions are made with the consensus of the child’s family. Most members of the American Academy of Pediatric Section of Critical Care did not support discontinuing therapy against the wishes of the patient’s parents, even if they agreed with the idea [24]. This reluctance may stem from the fear of potential lawsuits, which are common in the USA. Nonetheless, providing professional support to families during these difficult decisions should be a priority to avoid conflict. Potential conflicts between the therapeutic team and the patient’s family could be easily resolved if an objective mediator was involved in the communication process.

In our study, the majority of the respondents were intensivists who regularly make decisions about end-of-life care. In 2019, a survey conducted among Polish pulmonologists revealed that most respondents did not routinely talk to patients with advanced chronic obstructive pulmonary disease about end-of-life issues such as the need to make decisions about invasive mechanical ventilation, cardiopulmonary resuscitation, or the place of dying [25]. Similarly, it appears that Polish intensivists may not engage in these conversations as frequently as necessary, particularly in the ICU. However, our study suggests that intensivists are less influenced by the idealized vision of a miracle-working doctor and may find it easier to make decisions about discontinuing therapy. Regular contact with dying patients can help maintain some degree of autonomy from the emotional influences of the patient’s family or other specialties although it may also contribute to a type of burnout characterized by passivity, as our results prove.

Our research was limited by the representativeness of the study group for the entire medical community in Poland. The study group constituted less than 0.5% of all physicians in the country, and the selection of study participants was focused mainly on the southern voivodeships. The only criterion for inclusion was being a doctor. However, it is acknowledged that the issues of FT and the professional responsibility of doctors in the context of applicable legal acts in our country are important for fostering constructive discussions and developing actions that regulate the practice of such therapies and align with the expectations of both patients’ families and medical staff. Another limitation of our study is the absence of questions regarding opinions on the institution of a medical proxy, which warrants further research.

## Conclusions

The majority of the physicians who participated in the study supported the idea of not practicing FT, and this issue is well known to Polish medical staff. However, the obtained results confirmed, that intensivists had better knowledge of the discontinuation of FT, as well as more practical experience in caring for dying patients and they did not feel a heroic obligation to provide futile treatment by fighting to the end. Simultaneously the intensivists more frequently admitted to passivity in action, such as avoiding making decisions. We concluded, that further education and improved communication with the patient’s family are needed. Additionally, improving society’s awareness of the importance and role of the declaration of will and a medical proxy as an opportunity to influence end-of-life therapy may contribute to improving medical practice in this area, including an acceptance of refraining from FT. Medical doctors who have practical experience in the care of dying patients can contribute most to achieving this goal and preventing low-value care.

## Supplementary Material

ckae202_Supplementary_Data

## Data Availability

The data generated and analyzed in the current study are available from the corresponding author upon reasonable request.
