# Supplementary material for: Opinion of Polish doctors on the use of futile therapy
Source: Eur J Public Health. 2024 Dec 20;35(2):201–8. doi: 10.1093/eurpub/ckae202 (PMC11967861; doi:10.1093/eurpub/ckae202)
Supplement: ckae202_Supplementary_Data [file ckae202_supplementary_data.pdf]

A survey on the use and limitations of futile therapy

Dear Doctor,

I am sending you a survey regarding futile therapy (formerly called persistent therapy), which most often concerns patients hospitalized in intensive care units. This is a complex problem. We still don't know the opinion of Polish doctors on this subject, and the current level of awareness Polish society is also unknown.

Maintaining the functions of organs which does not bring any benefits to the patient and does not make it possible to achieve the assumed therapeutic goals is called futile therapy. Futile therapy prolongs the dying process and is related to the suffering of patients and their families, and the violation of human dignity.

Limiting futile therapy requires palliative treatment, i.e. ensuring the patient's comfort and providing care, nutrition, and hydration. The condition for limiting futile therapy is a consensus with the patient's family, preceded by a conversation between the therapeutic team and the patient's relatives, during which very detailed information about the patient's clinical condition and diagnostic and therapeutic decisions is provided.
Therefore, we would like to ask you to complete a short survey and express your opinion on this important topic by circling the most appropriate answer or writing it in the dotted space. You can select more than one answer regarding your opinion.

A. Personal data

A1. Age

A2. Gender Female Male

A3. Work experience (since graduation) …………… years

A4. Medical specialty

Work with children Yes ☐ No ☐

Work in ICU Yes ☐ No ☐

A. Anesthesiology and Intensive Care
B. Internal Medicine
C. Pediatrics
D. Neonatology
E. Surgery
F. Pediatric Surgery
G. Oncology
H. Pediatric Oncology
I. Cardiac Surgery
J. Other, specify ………………………………………………………………

A5. Workplace

A. University Hospital with Intensive Care Unit
B. Non-University Hospital with Intensive Care Unit
C. Hospital without Intensive Care Unit
D. only a Doctor's Office in a Clinic
E. Other, specify
……

A6. Religion

A. Catholic attending a mass every week
B. Catholic
C. Atheist
D. Other, specify
……

A7. Does the nature of your professional work involve contact with dying patients?

A. I don't think so, only several cases over the last few years
B. Several times a year
C. I have contact with such patients quite often, sometimes several times a month

A8. Were you a direct witness of the illness and death of a loved one?

Yes ☐ No ☐

B. Experience and opinion on futile therapy

B1. Have you encountered the concept of futile therapy in your professional practice?

A. In adults Yes □ No□
B. In children Yes □ No □

B2. Do you know the 'Guidelines regarding the ineffective maintenance of organ functions (futile therapy) in ICU patients incapable of giving informed statements of will', issued by the Polish Society of Anesthesiology and Intensive Therapy?

Yes □ No □

B3. Do you know the position of the Expert Group of Polish Episcopal Conference on Bioethics regarding futile therapy?

Yes □ No □

B4. Which medical procedures do you think should be limited (withheld or withdrawn) during futile therapy? (please tick the answer for each listed)

MEDICAL PROCEDURE	Definitely yes	Yes	No opinion	No	Definitely no
Patient intubation and mechanical ventilation					
Mechanical and pharmacological support of the circulatory system					
Extracorporeal membrane oxygenation (ECMO), extracorporeal carbon dioxide removal					
Renal replacement therapy					
Transfusion of blood products					
Antibiotic therapy					

B5. Have you ever talked to a patient and/or the patient's family about limiting futile therapy?

A. No, I did not have the opportunity due to the specificity of my specialization
B. No, despite the occurrence of such a clinical situation, I did not want to raise this topic
C. Yes, we had this conversation

B6. If the answer to question B5 is positive (Yes, we had this conversation), do you think you were prepared for such a conversation with the patient and/or the patient's family?

A. Definitely yes
B. Yes
C. No opinion
D. No
E. Definitely not

B7. In your opinion, what is the main reason for implementing/continuing futile therapy in adult patients? (more than one answer may be selected)

A. fear of talking to the patient/patient's family and their reaction
B. fear of legal liability for withdrawing or withholding treatment
C. heroic fight for life to the end, because this is the ethical duty of the doctor
D. fear of being accused by colleagues of the lack of professional ethics
E. fear of being accused by the patient's family of the lack of professional ethics
F. order/recommendation from the supervisor
G. passivity in action (colloquial term - laziness)

B8. In your opinion, what is the main reason for implementing/continuing futile therapy in newborns/children? (more than one answer may be selected)

A. fear of talking to the patient/patient's parents (family) and their reaction
B. fear of legal liability for withdrawing or withholding treatment
C. heroic fight for life to the end, because this is the ethical duty of the doctor
D. fear of being accused by colleagues of the lack of professional ethics
E. fear of being accused by the patient's family of the lack of professional ethics
F. order/recommendation from the supervisor
G. passivity in action (colloquial term - laziness)

B9. In your opinion, is the use of futile therapy a medical malpractice?

A. Definitely yes
B. Yes
C. No opinion
D. No
E. Definitely no

B10. Do you limit futile therapy in your practice?

A. Yes, but I omit this fact in the documentation
B. Yes, I note it in the documentation
C. No, because I don't want to be responsible for such a decision
D. No, because there is no such practice in the hospital ward where I work
E. This issue is not related to my specialization
F. Other………………………………………………………………………………………….

B11. In your opinion, what is the main reason for the family's refusal to limit futile therapy in their relatives? (more than one answer may be selected)

A. Not accepting the inevitability of death
B. Belief in the supernatural possibilities of treatment in the Intensive Care Unit
C. Lack of trust in doctors
D. All listed
E. Other, please specify……………………………………………………………………………..

B12. In your opinion, who should decide to limit futile therapy in adult patients? (please tick the answer for each listed)

**Who should decide to limit futile therapy in adult patients?**	**Definitely yes**	**Yes**	**No opinion**	**No**	**Definitely No**
Patient in a declaration of will/living will (decision expressed in writing before falling ill or in the presence of a witness)					
Doctors					

Head of the ward					
Nurses					
Patient's family					
Court					

B13. In your opinion, who should decide about limiting futile therapy in the case of a child?

**Who should decide about limiting futile therapy in the case of a child?**	**Definitely yes**	**Yes**	**No opinion**	**No**	**Definitely No**
Doctors					
Head of the ward					
Nurses					
Child's parents					
Guardianship Court					

B14. Do you think that economic aspects are important for the decision to continue futile therapy?

A. Definitely yes
B. Yes
C. No opinion
D. No
E. Definitely no
F. Economic aspects should not matter at all

B15. Are you convinced that the idea of limiting futile therapy is right?

A. Definitely yes
B. Yes
C. No opinion
D. No
E. Definitely no

B16. In your opinion, what would make the decision to limit futile therapy easier? (please tick the answer for each listed)

**What would make the decision to limit futile therapy easier?**	**Definitely yes**	**Yes**	**No opinion**	**No**	**Definitely No**

Unambiguous legal act					
Precise eligibility criteria for limiting therapy					
Education in this area (practical training, e.g. workshops)					
Patient's declaration of will/living will					

Other……
…

B17. If the problem of an incurable disease concerned you, would you like the futile therapy to be limited:

A. Definitely yes
B. Yes
C. No opinion
D. No
E. Definitely no

B18. If the problem of an incurable disease concerned you, who would decide to limit the futile therapy? (please tick the answer for each listed)

**If the problem of an incurable disease concerned you, who would decide to limit the futile therapy?**	**Definitely yes**	**Yes**	**No opinion**	**No**	**Definitely No**
Me in a declaration of will/living will					
Doctors					
Head of the ward					
Nurses					
Family					
Court					

Thank you very much for participating in the survey.
